# Hydrogen Sulfide Mitigates Kidney Injury in High Fat Diet-Induced Obese Mice

**DOI:** 10.1155/2016/2715718

**Published:** 2016-06-19

**Authors:** Dongdong Wu, Biao Gao, Mengling Li, Ling Yao, Shuaiwei Wang, Mingliang Chen, Hui Li, Chunyan Ma, Ailing Ji, Yanzhang Li

**Affiliations:** ^1^School of Medicine, Henan University, Kaifeng, Henan 475004, China; ^2^Kaifeng Central Hospital, Kaifeng, Henan 475000, China

## Abstract

Obesity is prevalent worldwide and is a major risk factor for the development and progression of kidney disease. Hydrogen sulfide (H_2_S) plays an important role in renal physiological and pathophysiological processes. However, whether H_2_S is able to mitigate kidney injury induced by obesity in mice remains unclear. In this study, we demonstrated that H_2_S significantly reduced the accumulation of lipids in the kidneys of high fat diet- (HFD-) induced obese mice. The results of hematoxylin and eosin, periodic acid-Schiff, and Masson's trichrome staining showed that H_2_S ameliorated the kidney structure, decreased the extent of interstitial injury, and reduced the degree of kidney fibrosis in HFD-induced obese mice. We found that H_2_S decreased the expression levels of tumor necrosis factor-*α*, interleukin- (IL-) 6, and monocyte chemoattractant protein-1 but increased the expression level of IL-10. Furthermore, H_2_S treatment decreased the protein expression of p50, p65, and p-p65 in the kidney of HFD-induced obese mice. In conclusion, H_2_S is able to mitigate renal injury in HFD-induced obese mice through the reduction of kidney inflammation by downregulating the expression of nuclear factor-kappa B. H_2_S or its releasing compounds may serve as a potential therapeutic molecule for obesity-induced kidney injury.

## 1. Introduction

Obesity, well known as a major public health issue worldwide, has been reported to be associated with many diseases, such as hypertension and type 2 diabetes mellitus [1–3]. Accumulating evidence suggests that obesity is also a major risk factor for the development and progression of chronic kidney disease (CKD) [3–5]. Along with the development of obesity, fast body weight gain increases tubular sodium reabsorption in the kidney, which in turn leads to renal vasodilation and glomerular hyperfiltration and eventually results in an increased glomerular filtration rate. Excess body weight gain may raise blood pressure, which can lead to increased renal blood flow. Consequently, these hemodynamic changes cause an increase of glomerular size accompanied by mesangial matrix expansion and podocyte injury [6–8]. Both hemodynamic and morphological changes, together with other factors, such as renal inflammation [9, 10], oxidative stress [9, 11], lipotoxicity [12, 13], insulin resistance [14], and fibrosis [15, 16], may result in renal dysfunction and ultimately lead to end-stage renal disease (ESRD).

Hydrogen sulfide (H_2_S) is an endogenous gaseous signaling molecule in mammalian tissues, which is enzymatically synthesized by cystathionine *β*-synthase (CBS), cystathionine *γ*-lyase (CSE), and 3-mercaptopyruvate sulfurtransferase (3-MST) [17–19]. All three enzymes are abundantly expressed in the kidney and are involved in renal H_2_S production [18, 20]. Accumulating evidence supports the roles of H_2_S in renal physiology and pathology [18–21]. Under physiological conditions, basal renal H_2_S regulates tubular functions and renal hemodynamics, including changes of renal blood flow, natriuresis, kaliuresis, glomerular filtration rate, and urinary excretion [18, 19, 22]. H_2_S can also act as an oxygen sensor in the renal medulla, where its accumulation in hypoxic conditions could restore oxygen balance by increasing medullary blood flow, reducing energy requirements for tubular transport, and directly inhibiting mitochondrial respiration [23, 24]. Because medullary hypoxia is a common pathologic feature of CKD, H_2_S deficiency may contribute to progression of CKD by limiting the important adaptive mechanism [23, 25].

Under pathological conditions, H_2_S plays a renoprotective role in the kidney. For instance, treatment with NaHS (a donor of H_2_S) could effectively attenuate ischemia/reperfusion-induced acute kidney injury by accelerating tubular cell proliferation and reducing superoxide formation and lipid peroxidation [26]. Importantly, the significant reduction of plasma H_2_S concentration and the downregulation of H_2_S-producing enzymes have been found in a rodent model of kidney injury and ESRD patients [23, 27]. H_2_S supplementation has been shown to reduce macrophage infiltration, glomerulosclerosis, and interstitial fibrosis as well as inhibit the blunt upregulation of adhesion molecules and inflammatory mediators [28]. Plasma H_2_S levels are also reduced in overweight subjects and patients with type 2 diabetes [29]. In obese mice, the biosynthesis of H_2_S was significantly reduced in the kidney after 16 weeks of feeding with a high fat diet [30]. Given the potent antioxidative, cytoprotective, and anti-inflammatory properties of H_2_S, we hypothesize that the application of exogenous H_2_S may protect against obesity-related kidney damage.

To test our hypothesis, a diet-induced obesity mouse model was employed in the present study. We found that the administration of NaHS could significantly improve kidney structure and function and suppress inflammation in the kidney of HFD-induced obese mice. Thus, H_2_S or its releasing compounds may possess therapeutic potential in treating obesity-related kidney disease.

## 2. Materials and Methods

### 2.1. Animals

The protocols for animal experiments were reviewed and approved by the Committee of Medical Ethics and Welfare for Experimental Animals of Henan University School of Medicine. Eight-week-old male C57BL/6JNju mice were purchased from the Nanjing Biomedical Research Institute of Nanjing University (Jiangsu, China). Mice were housed in individual ventilated cages in a temperature- and humidity-controlled environment on a 12-hour light/dark cycle with food and water* ad libitum*. The mice were fed either a low fat diet (LFD, 10% kcal as fat, Medicience Ltd., Jiangsu, China) or a high fat diet (HFD, 45% kcal as fat, Medicience Ltd., Jiangsu, China) for a total of 16 weeks. After 12 weeks of feeding, the LFD-fed mice were divided into the LFD group (6 mice) and the LFD + H_2_S group (6 mice); the HFD-fed mice were divided into the HFD group (6 mice) and the HFD + H_2_S group (6 mice). The mice from LFD and HFD groups received an intraperitoneal (i.p.) injection of saline; the mice from the LFD + H_2_S group and HFD + H_2_S group received an i.p. injection of NaHS (50 *μ*mol/kg/day, dissolved in saline) for 4 weeks. At the end of experiments, the mice were killed and the plasma was collected. Tissues were rapidly removed from the mice. The tissues were frozen in liquid nitrogen or embedded in FSC 22 frozen section compound (Leica, Buffalo Grove, IL, USA) or immersed in 4% neutral buffered formalin. Frozen tissues and plasma samples were stored at −80°C.

### 2.2. Histological Analysis

Kidney tissues were fixed in formalin, embedded in paraffin, and cut into 5 *μ*m thick sections. The sections were stained with a hematoxylin and eosin (HE) staining kit (Baibo Biotechnology Co., Ltd., Shandong, China), periodic acid-Schiff (PAS) staining kit (Baso Diagnostics Inc., Guangdong, China), and Masson's trichrome (MT) staining kit (Nanjing Jiancheng Bioengineering Institute, Jiangsu, China) according to the manufacturer's protocols. The PAS score is defined as follows: 0 = no deposits of PAS-positive material, 1 = up to one-third, 2 = one-third to two-thirds, and 3 = more than two-thirds of the glomerular cross section stain positive for PAS. Tubulointerstitial injury scores are defined as follows: 0 = no injury, 1 = less than 25%, 2 = 25–50%, 3 = 50–75%, and 4 = more than 75% [31]. Renal interstitial fibrosis (RIF) was scored from 0 to 3 as follows: 0 = absent (*n*_0_), 1 = less than 25% of the area (*n*_1_), 2 = 25–50% of the area (*n*_2_), and 3 = more than 50% of the area (*n*_3_). The RIF index was calculated according to the following equation: RIF index = (0 × *n*_0_ + 1 × *n*_1_ + 2 × *n*_2_ + 3 × *n*_3_)/(*n*_0_ + *n*_1_ + *n*_2_ + *n*_3_) × 100% [32]. All sections were scanned using an Olympus BX51 microscope (Olympus, Tokyo, Japan) and analyzed using Image J software (National Institutes of Health, Bethesda, MD, USA).

### 2.3. Biochemical Analysis

Kidney triglyceride (TG) was measured by enzymatic colorimetric method using a commercial kit according to the manufacturer's protocols (Applygen Technologies Inc., Beijing, China). Serum TG, cystatin C (Cys-C), creatinine (Cre), blood urea nitrogen (BUN), and kidney injury molecule- (KIM-) 1 were measured using a Roche Cobas 8000 automatic biochemical analyzer (Roche Diagnostics, Basel, Switzerland) according to the manufacturer's protocols. Tumor necrosis factor- (TNF-) *α*, interleukin- (IL-) 6, monocyte chemoattractant protein- (MCP-) 1, and IL-10 were determined using ELISA kits (Wuhan Elabscience Biotechnology Co., Ltd., Hubei, China) according to the manufacturer's protocols.

### 2.4. RNA Extraction and RT-PCR

Total RNA was isolated from kidney tissues using TRIzol reagent (Life Technologies, Rockville, MD, USA), treated with DNase I (Roche Applied Science, Mannheim, Germany), and purified using RNA clean-up kit (Cwbiotech, Beijing, China). One microgram of total RNA was applied for cDNA synthesis using a cDNA reverse transcription kit (Cwbiotech, Beijing, China). Primers were designed according to the primer design principles with Primer Premier 5.0 (Premier Biosoft, Palo Alto, CA, USA): TNF-*α*, forward 5′-GACGTGGAACTGGCAGAAGAG-3′ and reverse 5′-TTGGTGGTTTGTGAGTGTGAG-3′; IL-6, forward 5′-TAGTCCTTCCTACCCCAATTTCC-3′ and reverse 5′-TAAGACAATGGATCGGTCTAC-3′; MCP-1, forward 5′-CAGCCAGATGCAGTTAACGC-3′ and reverse 5′-GCCTACTCATTGGGATCATCTTG-3′; IL-10, forward 5′-GCTCTTACTGACTGGCATGAG-3′ and reverse 5′-CGCAGCTCTAGGAGCATGTG-3′; and 18S rRNA, forward 5′-AGAGTCGGCATCGTTTATGGTC-3′ and reverse 5′-CGAAAGCATTTGCCAAGAAT-3′. The PCR reactions were performed in a total volume of 20 *μ*L using the following thermal cycling parameters: 95°C for 1 min, 40 cycles of 94°C for 45 s, 58°C for 45 s, and 72°C for 1 min. The mRNA expression levels of the test genes were normalized to 18S rRNA levels.

### 2.5. Western Blot Analysis

Kidney tissues were homogenized in RIPA buffer (Sigma Chemical, St. Louis, MO, USA). Protein concentrations of kidney homogenates were measured with the BCA protein assay kit (Beyotime Institute of Biotechnology, Shanghai, China). The extracted proteins (40 *μ*g) were separated on SDS-PAGE gel and transferred to a nitrocellulose membrane. After blocking, the membranes were incubated with anti-TNF-*α* antibody (Beyotime Institute of Biotechnology, Shanghai, China), antinuclear factor-kappa B (NF-*κ*B), p65 antibody (Wuhan Boster Biotech Co., Ltd., Hubei, China), anti-NF-*κ*B p50 antibody (Wuhan Boster Biotech Co., Ltd., Hubei, China), anti-phospho-NF-*κ*B p65 (Ser536) antibody (Beyotime Institute of Biotechnology, Shanghai, China), and anti-*β*-actin antibody (Proteintech, Hubei, China). After washing, the membranes were incubated with horseradish peroxidase-conjugated secondary antibody (Beyotime Institute of Biotechnology, Shanghai, China). The reaction was visualized using an enhanced chemiluminescence system (Thermo Fisher Scientific, Rockford, IL, USA). Immunoblots were quantified by densitometry using Quantity One software (Bio-Rad, CA, USA).

### 2.6. Statistical Analysis

Data were presented as the means ± standard error of the mean (SEM). Differences between groups were analyzed by one-way analysis of variance (ANOVA) using SPSS 17.0 software, followed by Tukey's test. A *P* value of less than 0.05 was considered to be statistically significant.

## 3. Results

### 3.1. H_2_S Reduces Body Weight and TG Levels in HFD-Induced Obese Mice

**Figure 1:**
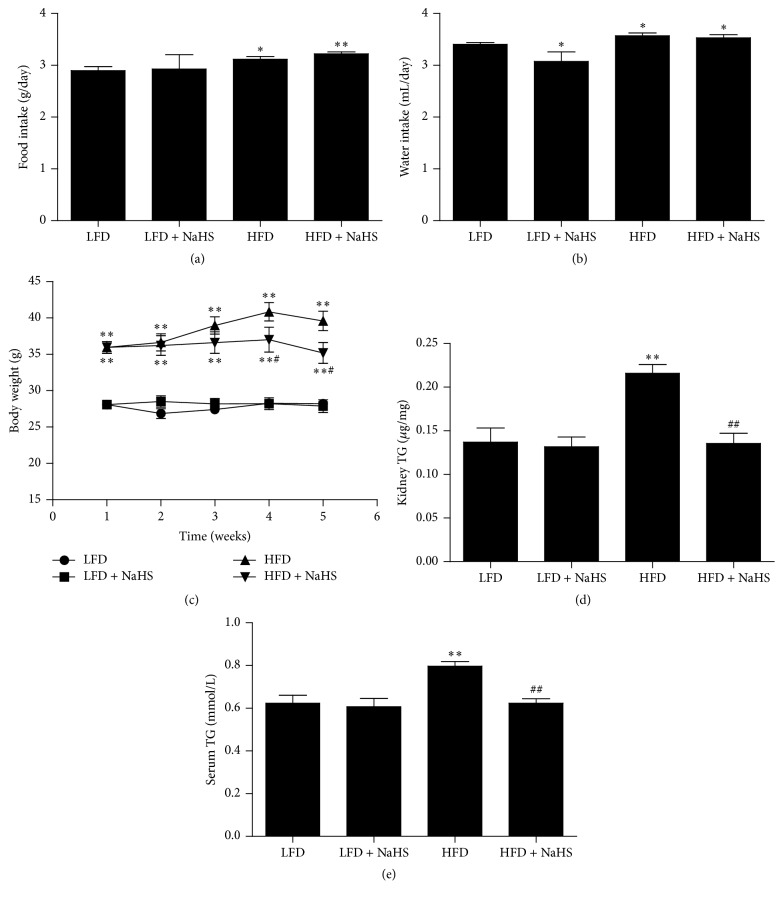
Effect of H_2_S on the body weight and TG levels of mice. ((a), (b)) Food intake and water intake were calculated. (c) The body weight of mice. (d) Kidney TG levels of mice. (e) Serum TG levels of mice. Values were presented as mean ± SEM (*n* = 6); ^*∗*^ *P* < 0.05; ^*∗∗*^ *P* < 0.01 compared with the LFD group; ^#^ *P* < 0.05; ^##^ *P* < 0.01 compared with the HFD group.

As shown in Figures 1(a)–1(c), in comparison with mice fed with LFD, HFD-fed mice exhibited increased food intake, water intake, and body weight. H_2_S treatment significantly decreased the body weight and TG levels of mice fed HFD, whereas H_2_S treatment did not change the body weight and TG levels of mice fed LFD (Figures 1(c)–1(e)).

### 3.2. H_2_S Treatment Improves the Kidney Function in HFD-Induced Obese Mice

**Figure 2:**
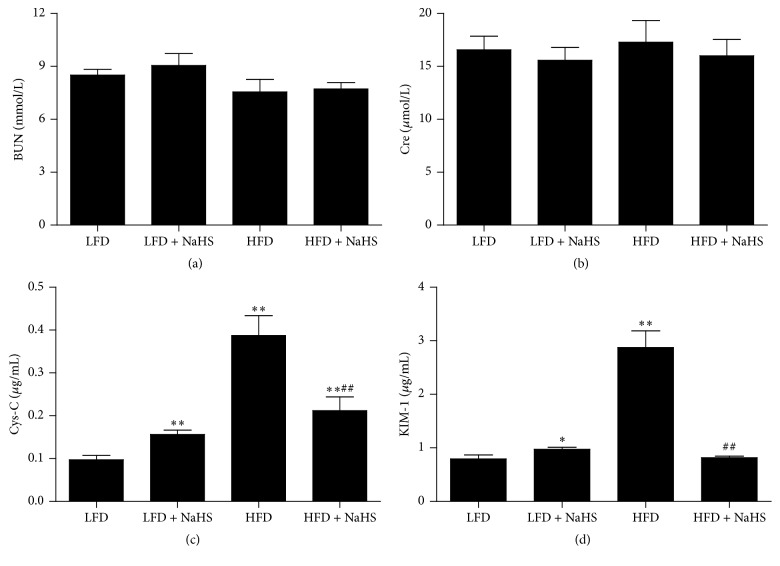
Effect of H_2_S on the kidney function of mice. The serum levels of BUN (a), Cre (b), Cys-C (c), and KIM-1 (d) were analyzed in each group of mice. Values were presented as mean ± SEM (*n* = 6); ^*∗*^ *P* < 0.05; ^*∗∗*^ *P* < 0.01 compared with the LFD group; ^##^ *P* < 0.01 compared with the HFD group.

In clinical practice, serum BUN and Cre are the most frequently used markers of kidney function [33, 34]. Cys-C and KIM-1 are considered sensitive and specific biomarkers in early kidney injury [33]. To investigate the effects of HFD and NaHS treatment on kidney function, the plasma levels of BUN, Cre, Cys-C, and KIM-1 in these mice were analyzed using biochemical methods. The results showed that there were no significant differences in plasma BUN and Cre levels between the LFD and LFD + NaHS group (Figures 2(a) and 2(b)). After 12 weeks of HFD feeding followed by 4 weeks of H_2_S treatment, the levels of plasma BUN and Cre were not changed, suggesting that HFD did not cause end-stage kidney injury. However, HFD induced 3- to 4-fold increases in plasma Cys-C and KIM-1 levels (Figures 2(c) and 2(d)), indicating that the kidney injury induced by HFD was in the early stage. After 4 weeks of H_2_S treatment, the plasma Cys-C and KIM-1 levels were significantly reduced, indicating that H_2_S has a therapeutic effect on early kidney injury induced by HFD.

### 3.3. H_2_S Ameliorates Kidney Structure in HFD-Induced Obese Mice

**Figure 3:**
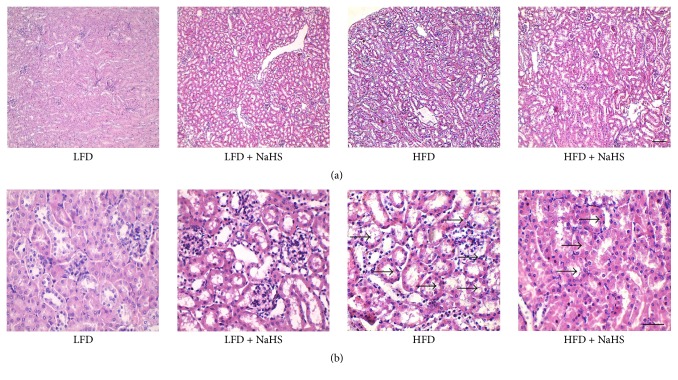
Evaluation of the kidney structure of mice using HE staining. Representative photographs of HE-stained kidney sections in each group of mice (*n* = 6/group). (a) Original magnification, ×100; scale bar = 200 *μ*m. (b) Original magnification, ×400; scale bar = 20 *μ*m.

To investigate the effect of H_2_S on kidney structure in HFD-induced obese mice, HE, PAS, and MT staining were performed. HE staining demonstrated that the kidney of the LFD control group showed clear tubular and glomerular structures and the tubular endothelial cells presented a good arrangement (Figures 3(a) and 3(b)). Upon HFD, the glomerular volume was increased, glomerular structure was unclear, Bowman's capsule was reduced, and sclerosis was observed in the glomerulus. The tubular endothelial cells swelled and became vacuoles, part of the tubular cavity expanded (Figures 3(a) and 3(b)). After NaHS treatment, the structure of the kidney was significantly improved, the glomerular volume was reduced, Bowman's cavity and the structure of the glomerulus became clear, and the tubular endothelial cells were arranged favorably (Figures 3(a) and 3(b)).

### 3.4. H_2_S Decreases the Carbohydrate Content and Interstitial Injury in HFD-Induced Obese Mice

**Figure 4:**
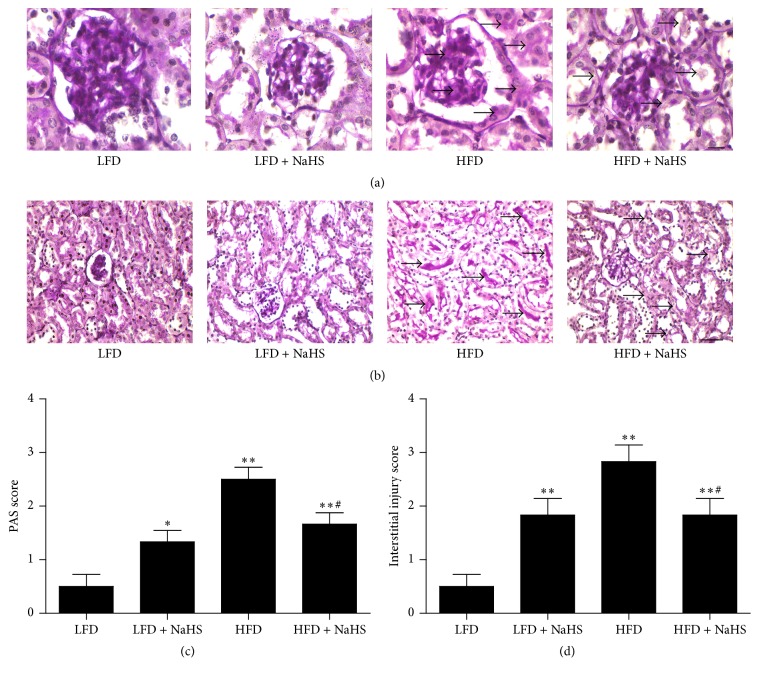
Evaluation of carbohydrate content in the kidney of mice using PAS staining. Representative photographs of PAS-stained glomerulus ((a) original magnification, ×1000; scale bar = 5 *μ*m) and renal tubular ((b) original magnification, ×400; scale bar = 20 *μ*m). Quantitative analysis of PAS-positive deposits (c) and interstitial injury (d) in each group of mice. Values were presented as mean ± SEM (*n* = 6); ^*∗*^ *P* < 0.05; ^*∗∗*^ *P* < 0.01 compared with the LFD group; ^#^ *P* < 0.05 compared with the HFD group.

PAS staining can be used to stain structures containing a high proportion of carbohydrate macromolecules, such as glycoprotein, glycogen, and proteoglycans [35]. Compared with the LFD, HFD increased the amount of PAS-positive staining in both glomerular and tubular structures (Figures 4(a)–4(c)), suggesting that the carbohydrate content was increased in the kidney of mice fed HFD. After NaHS treatment, the PAS-positive staining in both glomerular and tubular structures was reduced, demonstrating that NaHS treatment could effectively decrease the carbohydrate content in HFD-induced obese mice. Furthermore, as shown in Figure 4(d), HFD increased the interstitial injury, while NaHS treatment significantly decreased the interstitial injury.

### 3.5. H_2_S Ameliorates the Fibrosis of the Kidney in HFD-Induced Obese Mice

**Figure 5:**
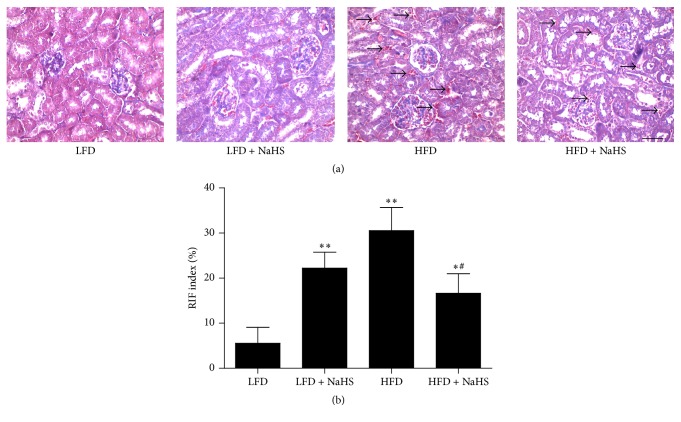
Evaluation of renal collagen deposition using MT staining (blue: collagen, pink: cytoplasm, and brown-black: nuclei). (a) Representative samples of MT staining (original magnification, ×400; scale bar = 20 *μ*m) in each group of mice. (b) The extent of the renal lesions is represented by the RIF index. Values were presented as mean ± SEM (*n* = 6); ^*∗*^ *P* < 0.05; ^*∗∗*^ *P* < 0.01 compared with the LFD group; ^#^ *P* < 0.05 compared with the HFD group.

Masson's trichrome is a three-color staining protocol used in histology, which produces red fibers, blue collagen, pink cytoplasm, and dark brown nuclei [32]. In the LFD control group, kidney tubular endothelial cells presented fullness of pink cytoplasm. By HFD, the amounts of blue collagen staining and red fiber staining were increased in kidney glomerular basement membrane and tubular interstitial area (Figure 5(a)), suggesting the accumulation of collagen and connective tissue fibers in the kidney. After NaHS treatment, the amounts of both blue collagen staining and red fiber staining were reduced (Figure 5(a)), and the RIF index was decreased (Figure 5(b)), indicating that H_2_S could play a role in ameliorating the fibrosis of kidney.

### 3.6. Treatment of NaHS Reduces Kidney Inflammation in HFD-Induced Obese Mice

**Figure 6:**
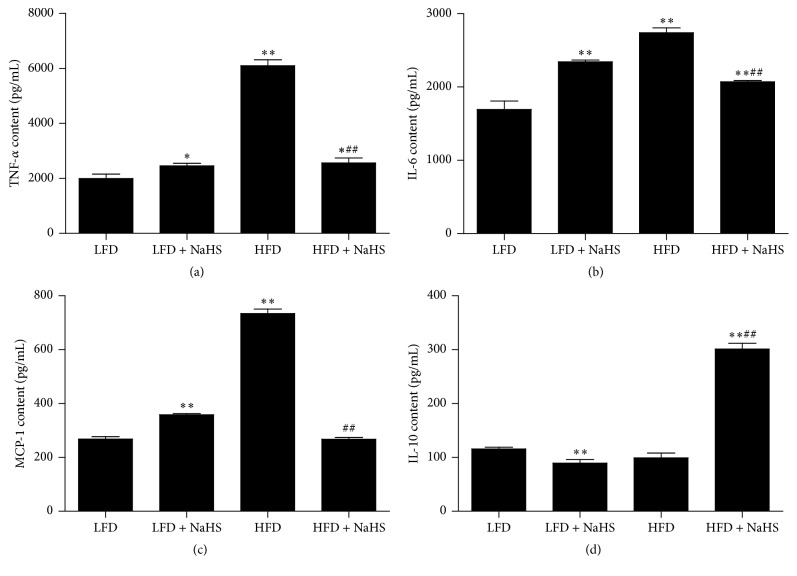
Effect of H_2_S on the cytokine levels in the kidney of mice was assayed using ELISA techniques. The expression levels of TNF-*α* (a), IL-6 (b), MCP-1 (c), and IL-10 (d) were analyzed. Values were presented as mean ± SEM (*n* = 6); ^*∗*^ *P* < 0.05; ^*∗∗*^ *P* < 0.01 compared with the LFD group; ^##^ *P* < 0.01 compared with the HFD group.

**Figure 7:**
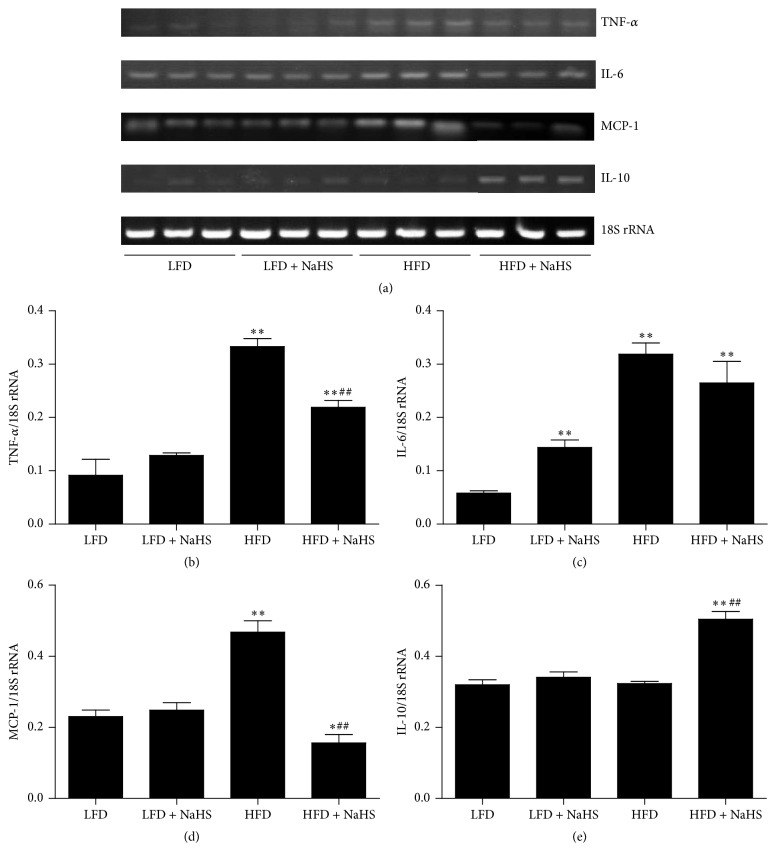
Effect of H_2_S on the cytokine levels in the kidney of mice was assayed using RT-PCR techniques. (a) The expression levels of TNF-*α*, IL-6, MCP-1, and IL-10 were measured by RT-PCR. 18S rRNA was used as an internal control. Bar graphs showed the quantification of TNF-*α* (b), IL-6 (c), MCP-1 (d), and IL-10 (e). Values were presented as mean ± SEM (*n* = 3); ^*∗*^ *P* < 0.05; ^*∗∗*^ *P* < 0.01 compared with the LFD group; ^##^ *P* < 0.01 compared with the HFD group.

Our results demonstrated that HFD induced injuries in kidney structure and function, and the injuries could be ameliorated by the administration of NaHS. To investigate the underlying mechanism, we examined kidney inflammation by analyzing the cytokine levels in the kidney using ELISA techniques. Compared with the LFD, HFD increased the expression levels of TNF-*α*, IL-6, and MCP-1 in the kidney (Figures 6(a)–6(c)). Treatment with NaHS significantly decreased the levels of TNF-*α*, IL-6, and MCP-1 (Figures 6(a)–6(c)), whereas it increased the IL-10 level (Figure 6(d)). The expressions of cytokine genes were further detected by RT-PCR. As shown in Figures 7(a)–7(d), compared with the LFD, HFD increased the gene expressions of TNF-*α*, IL-6, and MCP-1 in the kidney. Treatment of NaHS significantly decreased kidney TNF-*α*, IL-6, and MCP-1 gene expressions (Figures 7(a)–7(d)), whereas it increased kidney IL-10 expression (Figure 7(e)). These results indicated that H_2_S could reduce the kidney inflammation in HFD-induced obese mice.

### 3.7. H_2_S Reduces the Expression of NF-*κ*B in the Kidney of Obese Mice

**Figure 8:**
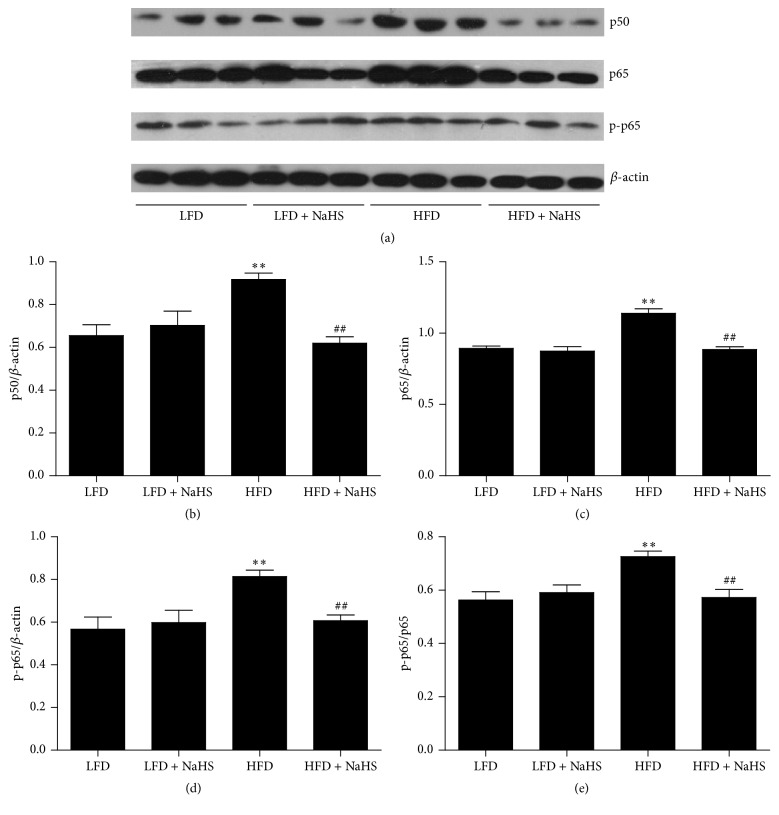
Effect of H_2_S on the protein level of NF-*κ*B in the kidney of mice. (a) The expression levels of p50, p65, and p-p65 were measured by Western blot. *β*-actin was used as an internal control. Bar graphs showed the quantification of p50 (b), p65 (c), p-p65 (d), and p-p65/p65 (e). Values were presented as mean ± SEM (*n* = 3); ^*∗∗*^ *P* < 0.01 compared with the LFD group; ^##^ *P* < 0.01 compared with the HFD group.

To investigate the mechanisms of NaHS on cytokine regulation, the expression level of NF-*κ*B was detected. NF-*κ*B is a transcription factor that plays a key role in the regulation of the expression of multiple cytokine genes involved in a variety of physiological processes [36]. The p50/p65 heterodimer is the most abundant and well understood of the NF-*κ*B dimers in most cells [37, 38]. We examined the protein expressions of p50, p65, and p-p65 in the kidneys of mice. As shown in Figures 8(a)–8(e), compared with the LFD, HFD increased kidney p50, p65, and p-p65 protein expressions and the p-p65/p65 ratio. Treatment with NaHS significantly decreased the protein expressions of p50, p65, and p-p65, as well as the ratio of p-p65/p65 in the kidney (Figures 8(a)–8(e)). The results indicated that HFD increased kidney inflammation through the upregulation of NF-*κ*B expression, and treatment with NaHS effectively reduced kidney inflammation by downregulating NF-*κ*B expression.

## 4. Discussion

H_2_S has been widely considered to be an endogenous gaseous signaling molecule, along with carbon monoxide and nitric oxide [17, 20, 21, 39]. Recently, an increasing number of studies suggest that H_2_S plays important and complex roles in renal physiology and pathophysiology, including the regulation of baseline hemodynamics and tubular properties [18, 19, 22], action as an oxygen sensor/transducer [28, 40], protection against renal I/R injury [21, 26, 41], attenuation of renal fibrosis [18, 42], and protection against glomerulosclerosis [28, 43]. Recent studies indicate that the H_2_S level was significantly reduced in overweight patients with type 2 diabetes and HFD-induced obese mice [29, 30]. In addition, there is increasing evidence that the obesity epidemic has coincided with an increased incidence of CKD [3, 8]. Therefore, we speculate that H_2_S deficiency may potentially contribute to the progression of CKD and the administration of exogenous H_2_S could mitigate CKD induced by obesity.

In the current study, we demonstrated that H_2_S significantly reduced the body weight and kidney TG levels of mice fed with HFD, suggesting that H_2_S plays an important role in lipid metabolism. However, the precise molecular mechanisms behind the role of H_2_S in the regulation of lipid metabolism need to be further investigated. A recent study showed that intrarenal arterial infusion of NaHS increased glomerular filtration rate, renal blood flow, urinary sodium, and potassium excretion, indicating that H_2_S is involved in the control of renal function [22]. Our data indicated that the kidney injury induced by HFD was in the early stage and H_2_S could maintain normal renal function by reducing the plasma levels of specific biomarkers in early kidney injury, such as Cys-C and KIM-1.

HE staining is the most common method used in the anatomic pathology diagnosis [44]. Our results showed that HFD increased the glomerular volume, reduced the Bowman's capsule, and resulted in glomerulosclerosis, suggesting that HFD could successfully induce renal injury. After treatment with H_2_S, the structure of the kidney was significantly improved in HFD-induced obese mice. It has been reported that the glomerular tuft area and the mesangial matrix area in mice fed with HFD tended to be larger than that in mice fed with LFD [45]. Similarly, we found that HFD increased the amount of PAS-positive staining in both glomerular and tubular structures, while H_2_S significantly reduced the PAS-positive staining in the kidney. Furthermore, H_2_S could decrease the interstitial injury in HFD-induced obese mice. A recent study revealed that H_2_S exhibited potent antifibrotic effects on obstructed nephropathy and inhibited the proliferation and differentiation of renal fibroblasts both* in vitro* and* in vivo* [18]. Our results indicated that HFD increased the red fiber staining in the glomerular basement membrane and tubular interstitial area and H_2_S effectively reduced the degree of renal fibrosis.

Obesity-related nephropathy is associated with regenerative cell proliferation, monocyte infiltration, and increased renal expressions of systemic proinflammatory TNF-*α*, IL-6, and MCP-1 [1, 9]. Similarly, our data indicated that HFD increased the expression levels of TNF-*α*, IL-6, and MCP-1 in the kidney. The administration of NaHS significantly decreased the expression levels of these proinflammatory cytokines. IL-10 is a Th_2_-type cytokine that is produced by a number of immunological cell types, including macrophages/monocytes, and it is a potent inhibitor of proinflammatory cytokines and chemokines [46, 47]. Notably, we found increased expression levels of IL-10. Furthermore, the gene expression of TNF-*α*, IL-6, MCP-1, and IL-10 exhibited similar changes. Thus, when the TNF-*α*/IL-6/MCP-1 results were contrasted with the IL-10 results, it appeared that the relative balance between proinflammatory and anti-inflammatory was “tipped” toward a proinflammatory state. The proinflammatory state played a mechanistic role in the progression of kidney injury. In contrast, treatment with H_2_S resulted in an anti-inflammatory state, which played an inhibitory role in the progression of kidney injury. However, it should also be noted that the administration of H_2_S increased the expression levels of TNF-*α*, IL-6, and MCP-1 and decreased the expression levels of IL-10 in LFD-fed mice. Furthermore, H_2_S increased the plasma levels of Cys-C and KIM-1 and enhanced the interstitial injury and fibrosis of the kidney in LFD-fed mice, which may be attributed to the opposite effect of H_2_S on inflammation [20, 48, 49]. Based on these studies, we hypothesize that H_2_S is able to exhibit an anti-inflammatory effect in HFD-fed mice and exert a proinflammatory effect in LFD-fed mice.

The NF-*κ*B network plays a crucial role in human health, and aberrant NF-*κ*B activation contributes to the development of a wide range of autoimmune, inflammatory, and malignant disorders, including atherosclerosis, multiple sclerosis, rheumatoid arthritis, inflammatory bowel diseases, and malignant tumors [36, 38, 50]. The NF-*κ*B family comprises five major members: c-Rel, p50, p52, p65 (Rel A), and Rel B [36, 38]. The p50/p65 heterodimer is the most important transcription factor of the canonical NF-*κ*B pathway and is commonly referred to as NF-*κ*B [38, 51]. A recent study demonstrated that high body adiposity could induce an inflammatory and proliferative microenvironment in the rat kidney [1]. Similarly, our data showed that HFD increased kidney p50, p65, and p-p65 protein expressions and the p-p65/p65 ratio, suggesting that HFD induced an inflammatory microenvironment in the kidneys of mice. However, the administration of NaHS significantly decreased the protein expressions of p50, p65, and p-p65, as well as the ratio of p-p65/p65 in the kidney. These results indicate that H_2_S could reduce kidney inflammation by downregulating NF-*κ*B expression.

In conclusion, our results suggest that H_2_S is able to reduce kidney lipids, improve kidney function, and reduce the interstitial injury and fibrosis of the kidney through the reduction of kidney inflammation by downregulating NF-*κ*B expression. Therefore, H_2_S or its releasing compounds may serve as a potential therapeutic molecule for obesity-induced kidney injury.
